# Time and Mind: A State-of-the-Art Perspective on Time Perception and Cognitive–Motor Interactions in Children and Adolescents with Cerebral Palsy

**DOI:** 10.3390/children12101283

**Published:** 2025-09-23

**Authors:** Giuseppe Accogli, Valentina Nicolardi, Mariangela Leucci, Luigi Macchitella, Greta Pirani, Maria Carmela Oliva, Antonio Trabacca

**Affiliations:** 1 Scientific Institute IRCCS “E. Medea”, Scientific Direction, 23842 Bosisio Parini, Italy; giuseppe.accogli@lanostrafamiglia.it (G.A.); valentina.nicolardi@lanostrafamiglia.it (V.N.); 2 Unit for Severe Disabilities in Developmental Age and Young Adults (Developmental Neurology and Neurorehabilitation), Scientific Hospital for Neurorehabilitation, Associazione “La Nostra Famiglia”, IRCCS “E. Medea”, 72100 Brindisi, Italy; mariangela.leucci@lanostrafamiglia.it (M.L.); luigi.macchitella@lanostrafamiglia.it (L.M.); greta.pirani@lanostrafamiglia.it (G.P.); mariacarmela.oliva@lanostrafamiglia.it (M.C.O.)

**Keywords:** cerebral palsy, time perception, executive functions, temporal processing, motor timing, pediatric neuropsychology, developmental disorders

## Abstract

**Highlights:**

**What are the main findings?**

• In children and adolescents with cerebral palsy, time perception difficulties are not fully explained by motor impairments and likely reflect broader cognitive–perceptual disruption.
• When studies minimize motor demands, selective time-perception deficits can still appear, indicating that timing can be affected independently of movement difficulties.

**What is the implication of the main finding?**

• Temporal perception should be treated as a distinct, clinically relevant domain in cerebral palsy, with consequences for daily functioning and development.
• Assessment and intervention should explicitly disentangle perceptual timing from motor execution, favoring motor-minimal paradigms to guide targeted rehabilitation.

**Highlights:**

**What are the main findings?**

• In children and adolescents with cerebral palsy, time-perception difficulties are not fully explained by motor impairments and may reflect broader cognitive–perceptual disruption.
• Studies that minimize motor demands sometimes reveal selective time-perception deficits, indicating that timing can be affected independent of movement difficulties.
• Temporal processing should be recognized as a distinct and clinically relevant domain in cerebral palsy, with consequences for daily functioning and development.
• Assessment and intervention should explicitly disentangle perceptual timing from motor execution and favor motor-minimal paradigms to guide targeted rehabilitation.

**Abstract:**

**Background:** Time perception (TP) is increasingly recognized as a key cognitive domain in children and adolescents with cerebral palsy (CP), yet existing studies are scarce, heterogeneous, and methodologically limited. **Objective:** To synthesize empirical evidence on TP in pediatric CP, distinguishing perceptual timing deficits from motor-based impairments and outlining putative cognitive mechanisms. **Methods:** Following PRISMA where appropriate, we systematically searched Scopus, Embase, and PubMed Central for studies on TP in individuals with CP under 18 years. Four studies met inclusion criteria. Risk of bias was appraised with STROBE, AXIS, and RoB 2. **Results:** Available evidence suggests that TP difficulties in CP are not solely due to motor dysfunction but also reflect broader cognitive–perceptual challenges. Studies using low-motor-demand tasks sometimes report intact TP, whereas tasks requiring overt movement often confound perceptual timing with execution demands. Intervention findings are mixed: time-related supports show promising but inconsistent effects on everyday time processing, while motor-focused timing training demonstrates limited impact on TP itself. However, conclusions are constrained by the small number of studies and variability in samples, tasks, and outcomes. **Conclusions:** TP should be considered a distinct, clinically relevant construct in pediatric CP. Future work should employ motor-minimal paradigms, report standardized CP classifications, and adopt longitudinal designs to isolate TP deficits and guide targeted interventions. Clarifying TP profiles may improve cognitive characterization and rehabilitation planning in CP.

## 1. Introduction

Cerebral palsy (CP) encompasses a group of conditions rather than a single disorder, characterized by varying degrees of severity and common developmental features. Rosenbaum and colleagues [1] define it as “a group of permanent disorders of the development of movement and posture, causing activity limitation, that are attributed to non-progressive disturbances that occurred in the developing fetal or infant brain.” These motor impairments often co-occur with disturbances in sensation, perception, cognition, communication, and behavior [2]. Cognitive comorbidities are common in CP: reviews indicate that about one in two children has measurable cognitive difficulties, with ~30–50% showing IQ < 70, and cognition is consistently lower in the presence of epilepsy and more severe motor impairment [3]. Executive functions are also markedly impaired, with a large overall difference versus typically developing peers (Hedges’ g ≈ −0.82; working memory g ≈ −0.92; inhibition g ≈ −0.82) [4]. European registry data further show clustering of neurodevelopmental comorbidities, e.g., autism spectrum disorder in 8.7% of children with CP, more frequent when intellectual disability and epilepsy co-occur [5].

Research on cognitive development in children with CP has gained increasing attention, emphasizing the need for systematic assessments and tailored interventions [2,6,7,8,9]. For instance, Bøttcher et al. [6] developed cognitive assessment protocols for CP, while Stadskleiv et al. [9] explored how motor impairments, brain injuries, and epilepsy contribute to cognitive variability. Fluss and Lidzba [8] emphasized that cognitive abilities are often underestimated in severe CP and overlooked in milder ones. Stadskleiv [2] called for comprehensive research on cognitive functioning across CP subtypes. Several cognitive domains have been explored in CP, including mathematical and visuospatial skills [10], working memory [11,12], learning and memory [13,14], attention [15,16], executive functions [17,18], and language [19,20].

While CP research has traditionally concentrated on motor impairments, emerging evidence highlights the importance of investigating time perception (TP) as a distinct cognitive domain [21,22]. TP refers to the ability to perceive and estimate durations, and it is closely linked to attention, memory, and sensory processing [23,24]. Several models have been proposed to explain the mechanism underlying TP. For example, the Pacemaker-Accumulator Model [25,26] points an internal clock-like mechanism that generates and accumulates pulses to estimate time, while the Dynamic Attending Theory [27] emphasizes attentional entrainment to temporal regularities in the environment.

Within this cognitive profile, TP is a cross-cutting capacity that scaffolds attention, working memory, and the temporal organization of behavior; consequently, TP deficits may compromise classroom learning (e.g., reading fluency), sustained attention, and everyday time management [28,29,30,31].

Our review is guided by two complementary accounts. The cognitive–perceptual view treats TP as explicit interval encoding/estimation modulated by attention, working memory, and decision stages, typically engaging supplemental motor area (SMA)–basal ganglia–prefrontal networks and yielding classic psychophysical signatures [32,33,34]. The motor view emphasizes timing in the service of action, where performance reflects both a central timekeeper and motor-implementation noise; high motor demands can therefore inflate apparent “timing” deficits [35,36]. Accordingly, we interpret studies using low-motor-load tasks as better isolating “pure” TP, whereas high-motor tasks are discussed in light of execution/prediction demands—consistent with CP findings showing near-typical performance in verbal/low-motor timing but larger errors when motor output is required [21,22,37].

**Figure 1:**
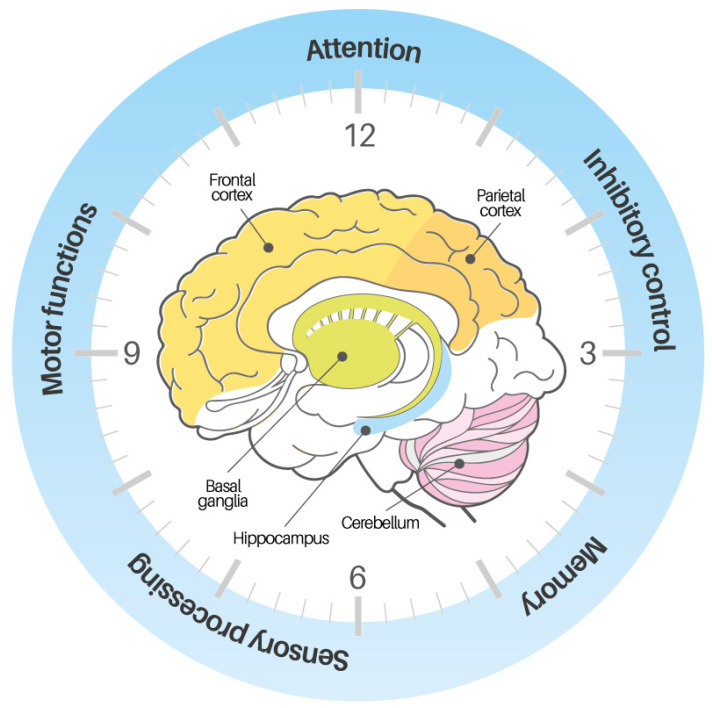
Neural Basis of Time Perception: Schematic of a distributed timing network. A core BG–SMA circuit supports interval timing and decision over durations; the cerebellum provides precise event timing and predictive calibration (especially sub-second and synchronization); prefrontal regions (DLPFC/right IFG) contribute attentional gating and working memory for timing judgments. The model integrates cerebellar event timing with BG–cortical accumulation/decision processes under PFC control.

Although TP and motor timing often interact and share overlapping neural substrates—such as SMA, basal ganglia, and cerebellum [32,35] (Figure 1)—they are conceptually distinct. TP involves the estimation of durations without requiring motor output, relying on attention, working memory, and perceptual processing [38,39]. In contrast, motor timing refers to the coordination of movements in time with external events, and depends more on sensorimotor integration [40,41]. Experimental paradigms such as passive interval estimation tasks or neuroimaging can dissociate these functions and are especially valuable in populations with motor impairments.

Converging evidence indicates that TP emerges from a core timing network comprising the SMA basal ganglia, and cerebellum, which interacts with task-specific cortical regions. Neurophysiology and imaging suggest both shared, core mechanisms (e.g., SMA–BG loops) and context-dependent computations across sensory and motor systems [33]. The cerebellum supports millisecond, event-based timing and prediction. Lesion and animal models show that the precise timing of conditioned responses depends on cerebellar cortex plasticity; when cerebellar mechanisms are disrupted, the temporal alignment between salient events degrades. In motor production, patients with cerebellar damage display increased temporal variability for discrete (event-segmented) movements but relative sparing for continuous movements—consistent with a cerebellar role in generating event timing signals rather than regulating continuous dynamics [32]. In humans, cerebellar activation is prominent for sub-second intervals and during synchronization to external rhythms, and perturbing the lateral cerebellum selectively worsens prediction of the temporal outcome of observed biological actions, underscoring its role in implicit timing and forward modeling [36]. Electrophysiology further places the SMA/M1–cerebellar system within the cascade of movement preparation and performance monitoring that scaffolds timed action [42]. The basal ganglia (BG) contribute to explicit timing across sub- and supra-second ranges via cortico-thalamic–striatal loops. During internally generated continuation (e.g., tapping without a metronome), the putamen–thalamus–SMA circuit is consistently engaged, aligning with models of an internal clock/accumulator whose dynamics are dopamine-sensitive (e.g., rate modulation). The striatal beat-frequency account formalizes how striatal ensembles could encode intervals through coincident cortical oscillatory inputs, providing a mechanistic bridge between psychophysics and circuitry [33]. Clinical evidence from movement disorders converges: Parkinson’s disease shows deficits in internally driven timing and bradykinesia that map onto BG circuit dysfunction, while perceptual timing can also be affected [36]. Notably, alternative interpretations emphasize BG contributions to decision and comparison stages within timing tasks, complementing clock-centric views [32]. Prefrontal systems (with inferior frontal and parietal partners) implement attention, working-memory gating, and decision operations that shape temporal judgments—especially at longer intervals. Right inferior frontal and parietal cortices are repeatedly implicated in explicit timing tasks; disruptive stimulation of prefrontal cortex alters perceived duration, consistent with a top-down control over accumulation/comparison processes [36]. More broadly, prefrontal cortex integrates cognitive and affective states (arousal, attention) that systematically bias subjective time, supporting models in which time is an emergent construct derived from distributed neural dynamics rather than a single dedicated “clock” [34].

Classic motor-timing work separates central timekeeping variability from motor implementation noise, explaining why tasks with high motor demands can inflate apparent timing deficits. This underscores the value of low-motor-load paradigms to probe “pure” TP [35]. Given frequent cerebellar/BG pathway involvement and executive-function vulnerabilities in CP, a mixed profile—relatively preserved timing in low-motor/verbal tasks but impairments when motor execution/prediction is required—fits the network account above and motivates motor-minimal assays of time perception in this population (and targeted interventions engaging cerebellar prediction and BG-SMA continuation).

### Terminology and Scope

**Time perception (TP).** We use *TP* to refer to sensory–cognitive mechanisms that encode and represent temporal information—such as duration, interval, temporal order and rhythm—independent of movement execution. Typical laboratory tasks include duration/interval discrimination, temporal bisection, temporal order judgment, and time estimation with a minimal motor response (e.g., a single keypress or a verbal answer) [23,24,25,26,27].

**Motor timing (MT).** *MT* refers to the generation and control of the temporal structure of movements: initiating, synchronizing and maintaining actions with temporal precision (e.g., paced or unpaced finger tapping, synchronization–continuation, rhythm reproduction). Conceptually and neurally it can overlap with TP (e.g., SMA, basal ganglia, cerebellum) but remains distinct in its primary motor demands [32,35,40,41].

**Time-Processing Ability (TPA).** *TPA* denotes a broader, functional construct relevant to everyday life. It includes TP together with time orientation (e.g., telling time, sequencing events) and time management (e.g., planning and executing activities within time constraints), typically assessed with pediatric, ecologically oriented instruments [43,44].

TP itself is composed of multiple components, including interval estimation, temporal discrimination, and synchronization with external rhythms [45]. Disentangling these components is key to accurately identifying which aspects of timing are impaired in children with CP. Disentangling these components is critical to understanding the nature of timing deficits in CP. Damage to structures such as the frontal cortex, BG, cerebellum, and hippocampus—which are implicated in both movement and timing—can affect temporal processing as well as motor control [46,47]. As a result, children with CP may present with timing difficulties that stem not only from motor execution problems but also from underlying cognitive–perceptual disruptions.

A major challenge in this area of research is to determine whether timing errors in CP are primarily due to impaired motor execution or to deficits in TP itself. For example, Olivier et al. (2015) [22] found that children with CP showed greater temporal variability in motor tasks, but this may reflect motor difficulties rather than pure perceptual impairments. However, this does not necessarily imply that TP is entirely preserved in children with CP. Given the role of sensory–motor integration in time estimation [23], motor dysfunction may indirectly influence cognitive timing.

Nevertheless, paradigms that minimize motor demands—such as passive estimation or neuroimaging—indicate that TP can be studied independently of movement [48,49,50].

TP impairments have been observed in various neurodevelopmental disorders [28,29,30,51], suggesting that targeted interventions for timing could also benefit children with CP. Cabezas and Carriedo (2020) [21] highlighted a link between temporal estimation and inhibitory control in children with CP, showing how higher-order executive functions influence timing abilities. Similarly, Giannotta et al. [31] emphasized the broader implications of TP dysfunction in neuropsychiatric populations, further supporting the relevance of this construct.

Although TP and motor timing often overlap in real-world tasks, understanding TP as a separate cognitive function is crucial. Accurate time estimation plays a central role in planning, anticipating consequences, and regulating behavior [52]. Its investigation should not be overshadowed by motor aspects alone, particularly in clinical populations like CP where the two domains may be confounded. Importantly, CP is a highly heterogeneous condition, with varying subtypes and comorbidities that can differentially affect cognitive and motor functioning [53,54,55]. In spastic CP, damage typically involves motor cortical areas and/or underlying white matter; in dyskinetic CP, lesions primarily affect BG; and in ataxic CP, cerebellar structures are chiefly involved [56,57]. Because the cerebellum plays a key role in fine temporal processing, children with ataxic CP might show unique patterns of TP impairment.

Recent work confirms that children and adolescents with CP show marked executive dysfunction across core domains [4] and randomized controlled trials that focus on executive functions can yield improvements (core executive functions and near-transfer), even if generalization is variable [58,59]. Beyond group-level impairment, daily life executive functioning relates to functional motor performance in unilateral CP [60]. Outside CP, converging evidence links TP with executive control: a neurodevelopmental review outlines tight TP–cognition relations [61], an EEG Go/No-Go study shows lower P300 to No-Go in low temporal-efficiency individuals (inhibitory control) [62], and a pilot RCT in ADHD finds TP remediation improves hot executive functions and behavior [63]. Within CP, the only study directly relating duration estimation to inhibitory control remains Cabezas & Carriedo (2020) [21]. Together, these findings strengthen a mechanistic between TP and executive functions account and motivate test it in CP using motor-minimal TP measures.

Given these complexities, studying TP in children and adolescents with CP as a distinct cognitive domain is essential. Future research should employ paradigms that minimize motor demands and focus on the perceptual and cognitive dimensions of time processing [48,49]. Such an approach can inform more accurate assessments and more targeted interventions.

Despite growing interest TP in CP, the available literature remains surprisingly limited. This knowledge gap not only constrains our understanding of cognitive profiles in developmental age, but also hinders the development of evidence-based and targeted rehabilitative interventions.

The aim of this review is therefore to examine TP in children and adolescents with CP as a key cognitive domain, clarifying how pure TP deficits can be distinguished from motor-based timing difficulties. In doing so, we synthesize current research findings on the neural and behavioral correlates of TP in CP, discuss methodologies that help isolate perceptual components from motor execution demands, and explore targeted interventions that may improve temporal processing as well as motor coordination. By emphasizing TP in its own right, we aim to deepen our understanding of the temporal dimension of CP and to inform more comprehensive approaches for assessment, rehabilitation, and overall quality of life in this population. Despite the limited number of studies, this review offers a state-of-the-art synthesis of current knowledge on the relationship between temporal perception and cognitive–motor interactions in children with CP. The striking scarcity of empirical research in this area underscores a critical gap in the literature and calls for increased scientific attention to temporal processing as an independent and clinically relevant domain.

## 2. Methods

**Figure 2:**
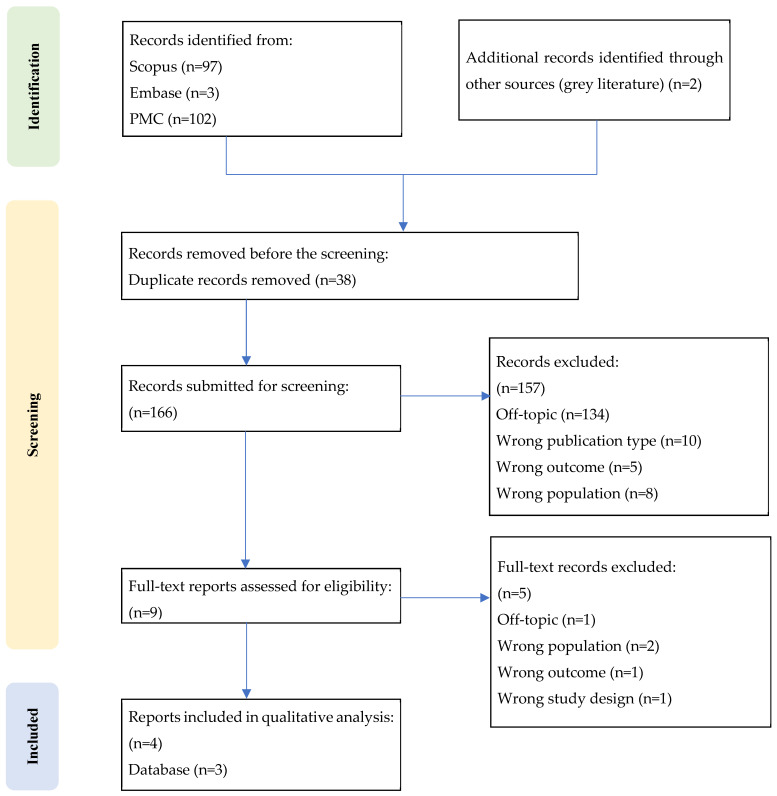
PRISMA flow diagram.

Although this review aims to provide a state-of-the-art overview of a poorly investigated field, we deliberately followed the PRISMA statement [64] to ensure methodological transparency and reproducibility. While state-of-the-art reviews are typically considered a form of narrative synthesis and are not required to adhere to PRISMA guidelines [65], we applied PRISMA where appropriate to increase the clarity and reproducibility of our methods. The search and selection process are summarized in Figure 2—PRISMA flow diagram-. The review protocol was not prospectively registered; nonetheless, the review adhered to PRISMA 2020, with eligibility criteria and analysis decisions defined a priori.

Two researchers independently conducted a literature search on Scopus, Embase, and PMC regarding TP, cognition, and motor timing in children with CP. Any conflicts between the two researchers were resolved with the intervention of a third party. In the database search, keywords related to TP, cognition, neuropsychological functions, and motor aspects were used, such as “infant cerebral palsy”, “temporal perception”, “temporal processing”, “cognition”, “cognitive impairment”, “motor control”, “motor timing”, etc. (Table 1). Studies were deemed eligible if they met the following criteria: written in English, featured in peer-reviewed journals, and consisted of original experimental articles (excluding reviews, overviews, and meta-analyses). Additionally, studies were included only if they focused on a pediatric population with CP (<18 years), excluding those involving adult populations or mixed groups (adult and children) unless separate analyses were provided for children. Given the extremely limited number of studies on this topic, we also considered original experimental studies on time-related cognitive processing in children with CP, including temporal perception, motor timing, and broader time-processing abilities. For the same reason, no chronological criteria were applied. The literature search was completed on 28 February 2025. To manage the article selection process, we utilized Rayyan, a web-based tool designed for systematic reviews. Rayyan autonomously assesses titles and abstracts, automatically removes duplicates, and reduces bias with its “blind” feature. Its text mining and labeling tools enhance workflow efficiency by enabling simultaneous abstract screening and full-text retrieval, streamlining the review process [66,67]. Data were extracted using a standardized form (population, intervention, outcomes, study design) and cross-checked among reviewers; Table 2 reports the general study characteristics, whereas participants’ age (means/ranges where available) and CP severity (GMFCS/MACS or author-defined scales) were extracted and are summarized narratively in the Results.

Sex/gender information was extracted when available; however, sex-stratified analyses were not feasible because the included studies did not report sex-specific outcomes and sample sizes were small.

**Figure 3:**
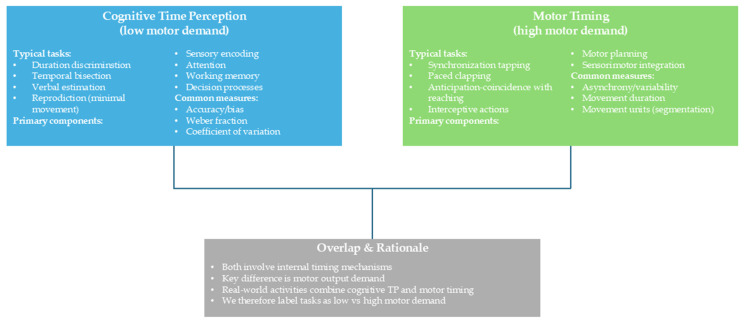
Conceptual distinction between cognitive time perception (low motor demand) and motor timing (high motor demand). Left: typical tasks (duration discrimination; temporal bisection; verbal estimation/reproduction), primary components (sensory encoding; attention; working memory; decision), and common measures (accuracy/bias; Weber fraction; coefficient of variation). Right: typical tasks (synchronization tapping/paced clapping; anticipation–coincidence with reaching; interceptive actions), primary components (motor planning and execution; sensorimotor integration), and common measures (asynchrony/variability; movement duration; movement units). Arrows indicate that both domains converge on shared timing mechanisms; our task labels reflect low vs. high motor demand.

A qualitative analysis was performed with tabulation of results and narrative comparison. Meta-analysis was not conducted due to data heterogeneity. To date, only four studies have been included based on the eligibility criteria. The characteristics of these studies are summarized in Table 2. The studies were organized according to methodological aspects such as their aim, experimental sample, control group, dependent variables and methods. To preserve this distinction, we annotated each task’s motor demand (low vs. high) and reported findings in relation to this classification, referring to low-motor-load paradigms when discussing “pure” time perception [32,35]. In line with prior timing literature, we distinguished cognitive time perception (perceptual timing tasks with minimal motor output) from motor timing (sensorimotor synchronization/anticipation tasks requiring overt movement) and labeled tasks as low vs. high motor demand (see Figure 3; e.g., [32,38,40,68]).

The risk of bias assessment was conducted using STrengthening the Reporting of OBservational studies in Epidemiology (STROBE checklist-cohort, case–control, and cross-sectional studies (combined)) [69] (Table 3) and the Appraisal tool for Cross-Sectional Studies (AXIS tool) [70] (Table 4). Two reviewers used STROBE for observational studies and AXIS for cross-sectional studies, with joint discussion to resolve discrepancies. For interventional studies, the RoB 2 tool [71] was used for the risk of bias assessment (Table 5).

## 3. Results

The initial search through databases yielded 204 articles, which were reduced to 166 after the removal of duplicates (duplicates = 38). Screening based on title and abstract led to the exclusion of 157 articles, leaving nine articles for full-text screening. The final articles included in the qualitative content analysis were four (Figure 2 and Table 2). While Table 1 summarizes the general characteristics of the included studies, details on age range and CP severity are synthesized narratively below because severity was not consistently classified across papers. For a schematic distinction between cognitive time perception and motor timing, see Figure 3.

Across the four included studies, pediatric participants with CP were between 6 and 16 years overall. In Cabezas & Carriedo (2020) [21], the CP group (n = 16) had a mean chronological age of 13.2 years (SD = 4.2) and was split between GMFCS II–III and GMFCS IV; GMFCS level V was explicitly excluded. In Olivier et al. (2015) [22], the CP sample (n = 11) ranged from 6 to 14 years and severity was rated on an author-defined four-level scale (3 low, 3 moderate, 2 severe, 3 very severe), with no GMFCS reported. In Johansson et al. (2014) [37], three cases with diplegic CP were aged 12–16 years and were reported at GMFCS III–IV and MACS II–IV. In Janeslätt et al. (2014) [43], the clinical sample was 6–11 years overall; the CP subgroup was not reported separately and CP severity was not classified with GMFCS/MACS. Taken together, the included CP cohorts predominantly represented moderate-to-severe motor impairment (GMFCS II–IV), and no study reported including GMFCS level V.

None of the included studies conducted sex/gender-stratified analyses of time-processing outcomes. Several reported only the sex composition of their samples—for example, Cabezas & Carriedo (2020) [21] reported the CP group as 37.5% girls and 62.5% boys and listed participant sex in Table 1; Janeslätt et al. (2014) [43] presented participants by age and gender and confirmed no baseline group difference in gender distribution; and Johansson et al. (2014) [37] reported participant sex within a three-case series—without any sex-specific outcome analyses.

The studies included in this review provide evidence that children with CP experience challenges in TP that extend beyond motor impairments and involve cognitive process. Of the four included studies, two primarily employed low-motor-load paradigms (Cabezas & Carriedo, 2020 [21]; Janeslätt et al., 2014 [43]—functional), one was mixed (Olivier et al., 2015 [22]), and one focused on high-motor-load timing training (Johansson et al., 2014 [37]).

Cabezas & Carriedo [21] investigated the relationship between inhibitory control and TP in children with CP, finding that deficits in inhibitory mechanisms were correlated with poorer performance in temporal estimation tasks. This suggests that higher-order executive functions contribute to TP difficulties in CP and that such impairments may not be solely attributable to motor dysfunctions. However, the nature of this relationship remains unclear. Impaired TP could result directly from executive dysfunction, or both TP and inhibitory control may be independently affected by underlying neural abnormalities. Future research should employ neuroimaging techniques to clarify whether inhibitory deficits precede TP impairments or arise in parallel.

Similarly, Olivier et al. [22] examined the interaction between TP and motor execution in anticipation-coincidence tasks, where children with CP had to predict the timing of an external stimulus using either a verbal estimation or a motor response. While they performed comparably to typically developing peers in the verbal condition, they exhibited greater temporal errors during motor responses. This finding supports the hypothesis that TP can be impaired independently of motor deficits, although movement difficulties may exacerbate the problem. It underscores the importance of separating pure TP impairments from motor timing dysfunctions.

In contrast, Janeslätt and colleagues [43] took a broader approach, assessing time-processing ability (TPA), which includes time perception, time orientation, and time management. In a randomized block, waiting-list design including children with developmental/intellectual disabilities (a subgroup with CP), the intervention group improved significantly more than the control group on time-processing ability (KaTid-Child) during the first period (Cohen’s d = 0.81, large). Parent-rated managing one’s time (Time-Parent scale) showed a medium effect (d = 0.68). After the waiting-list group later received the same intervention, they also improved. Fidelity checks indicated the program was delivered largely as planned. Collectively, these findings suggest that time aids can enhance both cognitive time-processing ability and everyday time management. Although the study did not isolate TP specifically, it provides indirect support for the cognitive nature of TP-related challenges and highlights the potential of interventions targeting time management.

Finally, Johansson et al. [37] explored the effects of synchronized metronome training (SMT) on motor timing and upper-limb coordination in children with diplegic CP. In a 4-week/12-session SMT case series (three children with diplegic CP), assessments at Pre, Post-training, and 6-month follow-up showed little change in motor timing per se, but two of three children exhibited lasting improvements in upper-limb kinematics (e.g., reduced movement duration and fewer movement units at shoulder, elbow, and wrist) and self-reported better hand/arm functionality and reduced muscle tone persisting at 6 months. One child showed minimal changes. These individualized yet durable kinematic and perceived-function gains support potential functional transfer from timing-focused training. These results suggest that motor-based interventions may not directly enhance TP, emphasizing the need for approaches that explicitly target cognitive timing mechanisms.

Across these studies, the evidence indicates that TP deficits in CP reflect not only motor difficulties but also challenges in cognitive processing—particularly involving executive functions and inhibitory control. The distinction between pure TP impairments and motor timing difficulties remains central to understanding temporal dysfunction in CP. While interventions such as time aids [43] appear promising in supporting broader time-related functions, motor-based training like SMT [37] shows limited impact on TP itself. Future research should continue to disentangle perceptual timing from motor execution constraints using experimental paradigms that isolate cognitive estimation processes.

## 4. Discussion

This review aimed to examine TP and motor timing in children with CP, emphasizing how TP difficulties interact with cognitive processes. The findings suggest that TP impairments in CP are not merely a consequence of motor dysfunction but also reflect broader cognitive challenges, particularly in executive functioning and inhibitory control. Research on TP in CP remains limited, with only four studies meeting the inclusion criteria, highlighting the scarcity of literature in this field. However, the included studies differ notably in their conceptual and methodological approaches. This pattern is consistent with the cognitive–perceptual vs. motor framework and with our low- vs. high-motor-demand annotation.

TP is closely tied to everyday functioning, including time orientation, planning, and managing daily routines. Evidence from our included studies indicates clear real-world consequences. In Cabezas & Carriedo [21], TP deficits were associated with inhibitory control, suggesting constraints on self-regulation in daily contexts (e.g., waiting, turn-taking, transitions). Olivier et al. [22] showed comparable accuracy in a low-motor verbal timing task but larger and more variable errors when timing required a reaching response—pointing to challenges in activities that combine timing with movement (e.g., getting ready on time, classroom transitions, PE tasks). In Janeslätt et al. [43] (mixed developmental sample including CP), a time-aids intervention yielded a large effect on time-processing ability and a medium effect on daily time management (parent ratings), emphasizing that caregiver/child reports capture ecological impact beyond standardized TP tests. Johansson et al. [37] further reported (in two of three cases) lasting improvements in upper-limb kinematics and perceived usability in daily activities after synchronized-metronome training, indicating functional transfer from timing-focused practice. Collectively, these findings support the ecological validity of TP deficits in CP and justify assessing both TP ability and everyday time management in clinical and educational settings.

TP shows features of a transdiagnostic construct across neurodevelopmental conditions. In children with CP, TP difficulties in this review clustered into cognitive TP (low motor demand) and motor timing (high motor demand), and related to domain-general functions and motor requirements: TP deficits were associated with inhibitory control [21] and became larger and more variable when timing required overt reaching [22]. Importantly, in a mixed developmental sample that included CP together with ADHD/ASD/ID, a time-aids program produced a large improvement in time-processing ability and a medium improvement in parent-rated daily time management [43], underscoring the everyday, transdiagnostic relevance of TP. In CP, synchronized-metronome training yielded durable gains in upper-limb kinematics and perceived function in two of three cases [37], suggesting potential functional transfer from timing-focused practice. Taken together, these patterns support a transdiagnostic view in which shared mechanisms (e.g., attention/working memory and temporal prediction) challenge TP across CP, ADHD, and ASD, while condition-specific constraints shape expression—most notably, motor demands amplify timing difficulties in CP, whereas timing problems in ADHD/ASD are often observable even in low-motor, cognitively loaded tasks. Clinically, this argues for assessing both cognitive TP and motor timing across diagnoses and for considering time-aids and timing-focused supports as potentially transdiagnostic interventions.

Clinically, timing deficits constrain classroom learning, attention, and everyday routines. In CP, poorer duration estimation co-occurs with inhibitory-control weaknesses [21], and timing errors are amplified when tasks require motor output compared with low-motor verbal variants [22], suggesting that executive and motor-implementation bottlenecks jointly degrade temporal control. Beyond CP, developmental evidence links atypical temporal sampling to phonological/reading difficulties in dyslexia [72] and robust perceptual/motor-timing impairment to attentional dysregulation in ADHD [73], situating TP within fronto-striato-cerebellar networks that support attention, working memory, and decision processes [52]. Functionally, interventions that externalize time (time-aids, the My Time program) improve time-processing ability and parent-rated daily time management/autonomy [43,74,75], whereas motor-focused timing training shows limited transfer to timing per se [37]. These data underscore the need to assess TP with low-motor-load paradigms and to combine executive-attention supports with environmental time-structuring to impact learning, attention, and day-to-day participation. Because this review is CP-focused, our comparison with ADHD/ASD is qualitative and grounded in the included mixed-sample evidence and shared timing mechanisms; a comprehensive synthesis of ADHD/ASD timing findings is beyond the present scope.

TP relies on executive control to allocate attention, keep temporal representations active, and suppress premature responses. Standard timing frameworks implicate prefrontal–basal ganglia–cerebellar circuits, predicting that weak inhibitory control increases response noise and bias, degrading TP even under low motor demand. In our CP set, poorer inhibition tracked with less accurate time estimation [21], and timing errors grew when tasks required reaching [22]; external time structuring improved time-processing ability and daily time management in a mixed sample including CP [43]. These converging findings support targeting executive functions—especially inhibition—alongside TP assessment and intervention in CP.

Beyond mechanisms, TP deficits carry tangible academic and social costs. In class, imprecise/variable timing undermines timed performance, time allocation, and transitions—consistent with the inhibition–TP association [21] and larger errors when timing requires reaching [22]. Socially, TP problems hinder waiting/turn-taking, synchronizing with peers, and daily routines; improvements in daily time management after time-aids in a mixed sample including CP [43] and better movement organization/usability after SMT in CP [37] support real-world relevance. These findings justify assessing TP and pairing it with simple supports (visual timers/schedules, extended time, structured transitions, low-motor-load tasks, and inhibition-focused scaffolds).

Our updated check found no CP studies directly testing the coupling between TP and executive function beyond Cabezas & Carriedo [21] (duration estimation ↔ inhibition). However, CP shows robust executive-function deficits [4], everyday executive functioning relates to bimanual performance in unilateral CP [60], and randomized controlled trials focused on executive functions report benefits on core executive abilities or near-transfer outcomes [58,59]. Outside CP, recent data connect TP with inhibitory control and hot executive functions [62,63] and frame TP within a neurodevelopmental model [61], supporting biological plausibility. We therefore recommend motor-minimal paradigms and integrated designs examining TP together with executive functioning CP (e.g., adding inspection-time or low-response-demand TP tasks) to test mediation/association models.

CP is heterogeneous with respect to motor type (e.g., spastic, dyskinetic, ataxic) and topography (hemiplegic, diplegic, quadriplegic), which plausibly modulates time-processing outcomes (standard classifications: [76,77]). In our set, representation was limited and uneven: Cabezas & Carriedo [21] included children with spastic or predominantly spastic CP (GMFCS I–IV; level V excluded); Johansson et al. [37] focused exclusively on diplegic CP with GMFCS III–IV/MACS II–IV; Olivier et al. [22] reported a non-standard 4-level severity score without GMFCS/MACS or CP subtype; and Janeslätt et al. [43] used a mixed diagnostic sample in which the CP subgroup was not analyzed separately. None of the included studies stratified TP outcomes by CP subtype. Consequently, apparent between-study differences may partly reflect underlying subtype and severity distributions. Practically, we encourage future TP work to prespecify CP motor type/topography, report GMFCS/MACS consistently, and (where feasible) stratify or adjust analyses by subtype—especially for tasks with higher motor demand where upper-limb involvement may differentially impact performance. Recognizing CP subtype heterogeneity is likely relevant for TP. In ataxic CP, cerebellar involvement would be expected to preferentially affect event-based millisecond and predictive timing [32,78]. In dyskinetic CP, basal ganglia/thalamic lesions may exert a greater impact on internally generated/interval timing and decision stages [33,79,80]. In spastic CP, periventricular white-matter/corticospinal injury may shift TP difficulties toward attention/working-memory contributions [2,81]. Direct pediatric TP-by-subtype evidence is limited; therefore, future studies should stratify by subtype/lesion pattern and report GMFCS/MACS.

Mechanistically, a distributed timing network yields phenotype-linked predictions for CP: ataxic CP—classically associated with cerebellar injury/malformations (vermis and hemispheres)—should show event-based, sub-second timing and predictive-calibration vulnerabilities; dyskinetic CP—typically linked to basal ganglia/thalamic lesions—should more often show distortions tied to internally generated timing and decision components. Prefrontal compromise (e.g., DLPFC/right IFG) can further amplify deficits via attentional gating and working-memory constraints. These circuit–phenotype correspondences are consistent with distributed accounts of time perception and justify the use of motor-minimal TP paradigms in CP to isolate perceptual timing from execution noise [32,33,34,35,36,42,80,82,83,84].

The conceptualization of TP and related constructs varies across studies. Cabezas & Carriedo [21] frame TP as a cognitive function closely tied to executive control, particularly inhibitory mechanisms. Olivier et al. [22] differentiate between cognitive time estimation and motor-dependent timing, emphasizing the impact of motor execution on perceived temporal errors. Johansson et al. [37] consider motor timing as a distinct process of synchronizing movements with external cues. Janeslätt et al. [43] propose a hierarchical model of TPA, distinguishing between TP, time orientation, and time management. This divergence highlights the need for integrative models that account for both cognitive and motor components of time processing in CP.

Hence, while Cabezas & Carriedo [21] explicitly explored the cognitive underpinnings of TP deficits, Olivier et al. [22], focused on TP in relation to motor execution, making it difficult to fully disentangle these aspects. The distinction between motor-dependent and purely cognitive TP remains methodologically challenging, a complexity further amplified by the intrinsic heterogeneity of CP itself [53,54,55]. Moreover, some studies included in this review examined broader TPA, which, while distinct from TP, are inherently connected. Although this review primarily focuses on TP and motor timing, we also considered TPA as a broader construct. Janeslätt et al. [43] examined time-processing ability and time management in children with various developmental conditions, including CP. While the study included a mixed clinical sample, its findings contribute to understanding cognitive aspects of time-processing in CP, particularly in daily life. The structured assessment of TPA adds ecological validity to the findings, even if it does not isolate TP from other components. The study found that children with disabilities, including CP, benefited from using time aids, highlighting the role of external supports in compensating for time-processing deficits. Although not CP-specific, the study supports the relevance of cognitive-based interventions for improving time-related functioning. However, the risk of bias assessment for Janeslätt et al. [43] raised several concerns, including CP-specific analyses, potential selection bias, and lack of randomization. Since time-processing deficits may vary across different neurodevelopmental conditions, the lack of CP-specific results limits the study’s applicability to this review’s primary focus. Sköld & Janeslätt [44] emphasized the distinction between TPA and daily time management, highlighting the importance of self-assessment tools to capture children’s subjective experience of time management in CP.

The interaction between TP and motor execution remains a crucial challenge in CP research. Olivier et al. [22] investigated coincidence-timing tasks, differentiating verbal time estimation from motor response-based timing. Their findings support the cognitive nature of TP, as children with CP performed similarly to typically developing peers in verbal estimation tasks but showed significant impairments in motor-dependent timing tasks. This suggests that TP may be preserved in some contexts but masked by motor deficits, reinforcing the importance of task design that isolates cognitive components. Conversely, Johansson et al. [37] examined the effects of SMT on motor timing and coordination in CP. While SMT improved movement organization, it had limited impact on TP itself, suggesting that motor-based interventions may not directly enhance cognitive time perception. These findings underscore the necessity of cognitive-focused interventions, aligned with the view that TP is influenced by attention, memory, and perceptual mechanisms[23,24]. They also confirm the relevance of temporal processing as a separate domain of investigation, one that remains at an early stage of empirical development.

The study by Olivier et al. [22] explored difficulties in coincidence-timing tasks among children with CP. Although it provided robust comparisons between CP children, typically developing peers, and adults, the AXIS Tool evaluation highlighted a lack of justification for the sample size and insufficient exploration of confounding variables, such as the severity of motor impairments. The STROBE Checklist assessment further revealed incomplete reporting of statistical methods and participant characteristics, limiting the study’s replicability. While the findings reinforce the impact of motor impairments on timing tasks, the binary separation between cognitive and motor components may underestimate the complexity of their interaction.

Children with CP risk having cognition—including TP—underestimated when motor, speech, or visual constraints depress task performance. To reduce bias, TP assessments should favor low-motor-load tasks and accessible response modes (eye-gaze, single-switch, augmentative and alternative communication), report accommodations transparently, and include all severity levels (avoid excluding GMFCS V). Triangulating standardized TP tasks with caregiver/child ratings of daily time management helps prevent misclassification and supports equitable access to interventions.

Collectively, these studies illustrate the challenges of investigating TP in children with CP. Although the STROBE Checklist showed that most studies adhered to essential reporting guidelines, the AXIS Tool evaluations highlighted consistent methodological weaknesses, particularly in addressing confounding factors within the context of study design and statistical analysis, as well as issues related to sample size and the use of validated measurement tools. The extreme paucity of available studies highlights the importance of this review as a state-of-the-art synthesis of current evidence, which may serve as a foundation for future work. Additionally, the heterogeneity in CP definitions and classifications [76,85] complicates research efforts, emphasizing the importance of standardized diagnostic criteria. From a methodological perspective, longitudinal studies are fundamental for understanding the evolution of motor and cognitive deficits in children with CP [86,87]. Monitoring prevalence trends and accompanying impairments over extended periods is crucial for understanding the progression of CP [88]. Longitudinal approaches also reduce investigator bias, enable comparison of multiple outcomes, and strengthen causal inference by controlling for confounders [89].

In conclusion, while existing studies provide important insights into TP in children with CP, their limitations in sample size, methodological rigor, and scope highlight the need for further research. A major limitation in current research is the lack of longitudinal studies tracking the development of TP over time. Given that both cognitive and motor functions evolve throughout childhood and adolescence, a longitudinal approach could reveal whether TP deficits remain stable, worsen, or improve with age and intervention. Additionally, studying CP populations over time could help determine if early cognitive and motor interventions mitigate later difficulties in TP. Future studies should incorporate larger cohorts, longer follow-up periods, and more nuanced methodologies to better capture the complexity of TP and its relationship with other cognitive and functional abilities. By leveraging both the AXIS Tool for methodological evaluation and the STROBE Checklist for reporting transparency, researchers can produce more reliable, generalizable, and clinically useful findings. Moreover, incorporating self-rating tools [44] could improve research on CP by complementing objective assessments and refining intervention strategies.

## 5. Conclusions

The current evidence highlights that TP difficulties in children with CP are influenced by both cognitive and motor factors, making it essential to distinguish between primary cognitive deficits and motor-related timing difficulties. While some interventions, such as assistive time management tools and SMT, have shown promising effects, results remain variable depending on the severity of CP, individual cognitive profiles, and methodological differences across studies. One of the key challenges in TP research in CP is the interaction between motor execution and cognitive processing. Some studies suggest that children with CP perform comparably to typically developing peers in verbal TP tasks but show significant impairments in motor-dependent timing tasks, indicating that TP deficits may not solely stem from motor dysfunction but also from higher-order cognitive processing difficulties, particularly in executive functions such as inhibitory control and working memory.

However, inconsistencies in research methodologies, small sample sizes, and non-standardized assessment tools limit the ability to draw definitive conclusions. Moreover, most studies rely on small sample sizes, heterogeneous participant groups, and cross-sectional designs, reducing the generalizability of findings. The lack of longitudinal data prevents a comprehensive understanding of how TP deficits evolve over time and how different interventions may impact both short-term and long-term outcomes.

Future research should focus on refining assessment methods to better isolate pure TP deficits from motor-related timing issues and develop targeted interventions that address both cognitive and motor challenges. Longitudinal studies will be essential to chart the developmental trajectory of TP impairments in CP. They could help identify critical periods where intervention is most effective and provide a more comprehensive picture of how TP difficulties interact with motor and cognitive development. To advance the field, studies should integrate standardized classification systems for CP severity, employ motor-free TP assessments, and explore novel intervention strategies tailored to the specific needs of children with CP. Addressing these research gaps will clarify the mechanisms underlying TP impairments and support the development of more effective, individualized rehabilitation strategies—ultimately improving daily functioning and quality of life, with likely benefits for classroom learning, sustained attention, and everyday time management, ultimately improving daily functioning and quality of life.

Recognizing whether difficulties reflect time perception per se or motor execution is critical for therapy planning. Low-motor-load timing assessments help identify TP deficits unconfounded by motor noise and thus guide tailoring [22,32,33]. When TP is impaired, combining environmental time structuring (time-aids) with executive-attention supports can improve time-processing ability and everyday time management/participation[21,43,74]. By contrast, motor-focused timing training shows limited transfer to timing itself, arguing for cognitive-attentional components within rehabilitation [37]. Finally, network accounts (SMA–basal ganglia–cerebellum) motivate targeting predictive timing and internally generated continuation with graded cueing when appropriate [32,33,36].

Despite its contributions to the understanding of TP in CP, this review has several limitations that must be acknowledged. Only four studies met the inclusion criteria, reflecting the scarcity of research on TP in CP. This limited evidence base restricts the scope of conclusions and precludes meta-analytical comparison. The included studies used different methodologies, sample characteristics, and outcome measures, making direct comparisons difficult. Such heterogeneity reduces the potential for synthesis within a unified conceptual framework. Due to differences in study designs and outcome measures, a quantitative meta-analysis was not performed. A statistical aggregation of findings could have provided more definitive conclusions on the magnitude and variability of TP impairments in CP. The reviewed studies included participants with different CP subtypes and severity levels, but few studies provided a standardized classification using tools like the Gross Motor Function Classification System (GMFCS). This variability limits the interpretability of findings regarding how TP deficits vary across clinical profiles. However, the very paucity of studies reinforces the need for systematic investigation. The strength of this review lies in offering a structured, state-of-the-art synthesis of a neglected yet promising field of inquiry. Moreover, beyond inhibitory control, cognitive flexibility (set-shifting/updating) may also influence TP tasks; although none of the included studies assessed it, CP cohorts show flexibility impairments [4,90].

Because none of the included studies analyzed outcomes by sex/gender—and samples were small—we could not evaluate potential sex-related differences in TP in CP. Future studies should prespecify and report sex/gender-disaggregated results.

Collectively, the highlighted limitations underscore the urgency of developing standardized research protocols for studying TP in CP populations.

## Figures and Tables

**Table 1:** Search strings used in the review. An English-language filter was applied.

Database	String
Scopus	(“Cerebral palsy” OR “Infant cerebral palsy” OR “Pediatric cerebral palsy” OR “Paediatric Cerebral Palsy” OR “Child* with cerebral Palsy”) AND (“Temporal Processing” OR “Time Processing” OR “Time Perception” OR “Temporal cognition”) AND (“Cognition” OR “Cognitive impairment” OR “Cognitive function” OR “Cognitive development” OR “Neuropsychological impairment” OR “Neuropsychological function” OR “Neuropsychological development” OR “Neuropsychological outcome” OR “Executive function*” OR “Inhibitory control” OR “Memory” OR “Working memory” OR “Attention” OR “Self-regulation” OR “Problem-solving” OR “Decision-making” OR “Learning ability” OR “Memory processing” OR “Processing speed” OR “Intellectual function*” OR “Mental flexibility” OR “Metacognition”) AND (“Motor control” OR “Motor planning” OR “Motor timing” OR “Movement timing” OR “Motor coordination” OR “Temporal motor control”)
Embase	(‘cerebral palsy’/exp OR ‘cerebral palsy’ OR ‘infant cerebral palsy’ OR ‘pediatric cerebral palsy’ OR ‘paediatric cerebral palsy’ OR ‘child* with cerebral palsy’) AND (‘temporal processing’/exp OR ‘temporal processing’ OR ‘time processing’ OR ‘time perception’/exp OR ‘time perception’ OR ‘temporal cognition’) AND (‘cognition’/exp OR ‘cognition’ OR ‘cognitive impairment’/exp OR ‘cognitive impairment’ OR ‘cognitive function’/exp OR ‘cognitive function’ OR ‘cognitive development’/exp OR ‘cognitive development’ OR ‘neuropsychological impairment’/exp OR ‘neuropsychological impairment’ OR ‘neuropsychological function’/exp OR ‘neuropsychological function’ OR ‘neuropsychological development’/exp OR ‘neuropsychological development’ OR ‘neuropsychological outcome’ OR ‘executive function*’ OR ‘inhibitory control’/exp OR ‘inhibitory control’ OR ‘memory’/exp OR ‘memory’ OR ‘working memory’/exp OR ‘working memory’ OR ‘attention’/exp OR ‘attention’ OR ‘self-regulation’/exp OR ‘self-regulation’ OR ‘problem-solving’/exp OR ‘problem-solving’ OR ‘decision-making’/exp OR ‘decision-making’ OR ‘learning ability’/exp OR ‘learning ability’ OR ‘memory processing’ OR ‘processing speed’/exp OR ‘processing speed’ OR ‘intellectual function*’ OR ‘mental flexibility’/exp OR ‘mental flexibility’ OR ‘metacognition’/exp OR ‘metacognition’) AND (‘motor control’/exp OR ‘motor control’ OR ‘motor planning’/exp OR ‘motor planning’ OR ‘motor timing’/exp OR ‘motor timing’ OR ‘movement timing’ OR ‘motor coordination’/exp OR ‘motor coordination’ OR ‘temporal motor control’)
PMC	(“Cerebral palsy”[All Fields] OR “Infant cerebral palsy”[All Fields] OR “Pediatric cerebral palsy”[All Fields] OR “Paediatric Cerebral Palsy”[All Fields] OR “Child* with cerebral Palsy”[All Fields]) AND (“Temporal Processing”[All Fields] OR “Time Processing”[All Fields] OR “Time Perception”[All Fields] OR “Temporal cognition”[All Fields]) AND (“Cognition”[All Fields] OR “Cognitive impairment”[All Fields] OR “Cognitive function”[All Fields] OR “Cognitive development”[All Fields] OR “Neuropsychological impairment”[All Fields] OR “Neuropsychological function”[All Fields] OR “Neuropsychological development”[All Fields] OR “Neuropsychological outcome”[All Fields] OR “Executive function*”[All Fields] OR “Inhibitory control”[All Fields] OR “Memory”[All Fields] OR “Working memory”[All Fields] OR “Attention”[All Fields] OR “Self-regulation”[All Fields] OR “Problem-solving”[All Fields] OR “Decision-making”[All Fields] OR “Learning ability”[All Fields] OR “Memory processing”[All Fields] OR “Processing speed”[All Fields] OR “Intellectual function*”[All Fields] OR “Mental flexibility”[All Fields] OR “Metacognition”[All Fields]) AND (“Motor control”[All Fields] OR “Motor planning”[All Fields] OR “Motor timing”[All Fields] OR “Movement timing”[All Fields] OR “Motor coordination”[All Fields] OR “Temporal motor control”[All Fields])

**Table 2:** Summary of the literature research based on inclusion criteria. Four studies met the inclusion criteria. The studies were summarized based on the author, year, title, aim, experimental group, control group, dependent variable, use of functional scale to classify CP severity, comorbidities, methods used, results, and limitations.

Authors	Year	Title	Aim	Experimental Sample	Control Group	Dependent Variable	Use of Functional Scale	Comorbidities	Methods	Results	Limitations
Janeslätt et al. [43]	2014	Evaluating intervention using time aids in children with disabilities	To evaluate a complex intervention (time aids) for children (6–11 years) with intellectual/developmental disabilities, focusing on time-processing ability and daily time management.	Mixed sample (N = 47) including ADHD, ASD, mild/moderate ID, spina bifida, CP; assigned to Intervention group (n = 22) or Control group (n = 25).	Yes, Control group (Waiting List design); after 6 months, the control group also received the intervention.	Time-processing ability (KaTid-Child) & managing one’s time (Time-Parent scale). Motor demand: Functional-low.	No GMFCS or MACS usage reported.	Multiple diagnoses in sample (ADHD, ASD, ID, spina bifida, CP).	Randomized Block + Waiting List design; 6-month intervention with time aids; pre-/post-measures of time-processing ability and parent-rated daily time management.	Intervention group improved more than controls in time-processing ability and daily time management; effect sizes were large/medium. After controls received the same intervention, they also improved.	Sample includes multiple diagnoses; no separate analysis for CP only; no standardized CP severity measure; fairly small subgroups.
Cabezas & Carriedo **[21]**	2020	Inhibitory control and temporal perception in cerebral palsy	To study whether children/adolescents with CP show deficits in inhibitory control and duration estimation, and to test whether inhibitory difficulties affect time estimation.	CP group (N = 16) with spastic or predominantly spastic CP; mental ages between 5.5–10 yrs.	Yes, Typically developing controls (N = 16), matched by mental age.	Inhibitory control task performance (Stroop-like tasks, etc.) and accuracy in duration estimation tasks. Motor demand: Low.	Yes GMFCS levels I–IV used.	CP can involve various comorbidities; not specified in detail.	Two inhibitory control tasks plus two interval-estimation tasks; compared CP group vs. typically developing controls.	CP group showed lower performance in both inhibition and temporal perception vs. controls; found a relationship between inhibition and time estimation.	Sample size relatively small (N = 16 CP); wide age range; only spastic CP forms included. Nonetheless, they used GMFCS classification, excluding level V.
Johansson et al.** [37]**	2014	Timing training in three children with diplegic cerebral palsy: short- and long-term effects on upper-limb movement organization and functioning	To explore effects of a 4-week/12-session synchronized metronome training (SMT) program on motor timing and upper-limb function in three children (diplegic CP). Assess immediate and 6-month outcomes.	Single-case design with three children (ages 12–16) diagnosed with diplegic CP (DCP).	No formal control group.	Motor timing (deviation from metronome beat), upper-limb kinematics, subjective arm/hand functioning. Motor demand: High.	Yes: GMFCS & MACS levels detailed for each child.	DCP is the primary diagnosis; no additional comorbidities described.	Kinematic assessment of a goal-directed upper-limb task (3D motion capture), synchronized metronome training tasks (Interactive Metronome^®^). Pre-training, post-training (1 week), and follow-up (6 months).	Two of the three children showed notable improvements in movement organization, speed, smoothness, and subjective hand/arm control lasting at 6 months post. One child had minimal changes.	Very small sample (3 children); no control group; individual outcomes; caution generalizing results; but GMFCS & MACS usage is explicitly reported.
Olivier et al.** [22]**	2015	Cognitive and motor aspects of a coincidence-timing task in Cerebral Palsy children	To assess how children with CP handle anticipation-coincidence tasks, comparing purely cognitive (verbal timing) and motor (reaching) responses.	CP group (N = 11, ages 6–14); severity rated on a custom 4-level scale (1 = low to 4 = high). Plus 51 typically developing children (6–13 yrs) and 13 healthy adults.	Two groups: 51 typically developing children and 13 healthy adults.	Timing errors (under-/overestimation of an event), measured in verbal vs. motor tasks. Motor demand: Mixed (verbal = Low; reaching = High).	No GMFCS or MACS used; authors mention a custom severity scale.	Primarily CP (motor deficits), no other comorbidities reported.	Participants performed a “coincidence-timing” task triggered by a musical cue. In the “verbal” condition, they responded vocally; in “motor” condition, they reached for a target. Accuracy/variability in timing errors was measured in each condition.	CP children performed similarly to controls in purely verbal timing but had larger and more variable errors in the motor task; they partially compensated for their motor deficits but still showed worse performance than typically developing children or adults.	Small CP sample (N = 11); use of a non-standard 4-point severity scale instead of GMFCS/MACS; results focus on timing performance, do not systematically address functional motor classification.

**Table 3:** STROBE checklist risk of bias assessment for Cabezas & Carriedo, 2020 [21]; Johansson et al., 2014 [37]; Olivier et al., 2015 [22].

Recommendation	Cabezas & Carriedo, 2020 [21]	Johansson et al., 2014 [37]	Olivier et al., 2015 [22]
(a) Indicate the study’s design with a commonly used term in the title or the abstract. (b) Provide in the abstract an informative and balanced summary of what was carried out and what was found.	Study design mentioned as experimental tasks involving visual and auditory stimuli.	Study described as an intervention exploring motor timing in CP children.	Coincidence-timing task study involving children with cerebral palsy.
Explain the scientific background and rationale for the investigation being reported.	Study investigates time perception in children with CP.	Background provided on timing training and motor synchronization in CP.	Rationale focuses on cognitive and motor aspects of timing in CP children.
State specific objectives, including any prespecified hypotheses.	To assess time perception in CP vs. typically developing children. The study hypothesized that children with CP would perform worse in temporal estimation and inhibitory control, especially under high interference. It also expected a link between inhibitory control and temporal processing	To evaluate timing ability and retention in CP children. Children with more severe diplegic CP will show improvements in timing ability after 4 weeks of Interactive Metronome. IM training will lead to long-term retention effects in spatio-temporal movement organization.	To dissociate cognitive and motor components of timing tasks. The authors hypothesized that temporal estimation in CP children should not be different with respect to healthy children and adults when simple motor response was involved. Conversely, it was expected that when the coincidence-timing task required more complex movement execution, coincidence-timing was altered with respect to healthy subjects.
Present key elements of study design early in the paper.	Study tasks presented via computer using controlled stimulus duration.	Kinematic analysis used to assess timing ability.	Musical sequence and motor response analyzed in CP children.
Describe the setting, locations, and relevant dates, including periods of recruitment, exposure, follow-up, and data collection.	Children with CP recruited via clinical records.	Participants recruited from a rehabilitation center in Sweden.	CP children recruited based on ability to perform coincidence-timing.
(a) Give the eligibility criteria, and the sources and methods of selection of participants. (b) For matched studies, give matching criteria and number of exposed and unexposed.	Study recruited children with CP and typically developing peers from clinical centers.	Eligibility criteria: diagnosed with diplegic CP, undergoing rehabilitation.	Selection criteria included ability to perform auditory-based timing task.
Clearly define all outcomes, exposures, predictors, potential confounders, and effect modifiers. Give diagnostic criteria, if applicable.	Outcome measures: reaction time, temporal perception accuracy.	Measures: kinematic movement trajectories, timing errors.	Coincidence-timing accuracy and motor response times assessed.
For each variable of interest, give sources of data and details of methods of assessment (measurement).	Methods included controlled experimental design with standardized stimuli.	Movement tracking and motor timing analysis using optoelectronic system.	Auditory–motor synchronization task measured precision of responses.
Describe any efforts to address potential sources of bias.	Efforts included matched control group to compare CP vs. typically developing children. The document does not explicitly describe any strategies to address potential biases in the study. While participant characteristics and measurement methods are mentioned, no specific actions to mitigate selection or measurement bias are reported. Participants were chosen from a specific population which may limit generalizability.	Bias minimized by individualized training protocols and repeated measures. No explicit strategies to address potential bias were discussed.	Addressed by controlling for variability in auditory and motor responses. No strategies for addressing potential bias explicitly discussed.
Explain how the study size was arrived at.	Sample size: 16 CP and 16 control participants. No sample size calculation.	Small sample of 3 CP children with detailed movement tracking. No sample size calculation.	Study included 15 CP children, 51 healthy children, and 13 adults. No justification for the small sample size provided.
Explain how quantitative variables were handled in the analyses. If applicable, describe which groupings were chosen and why.	Quantitative variables included response times and error rates.	Timing errors analyzed with precision metrics and kinematic models.	Response accuracy and movement execution times assessed quantitatively.
(a) Describe all statistical methods, including those used to control for confounding. (b) Describe any methods used to examine subgroups and interactions. (c) Explain how missing data were addressed. (d) If applicable, explain how loss to follow-up was addressed. (e) Describe any sensitivity analyses.	Statistical methods: ANCOVA, correlation and regression analysis between inhibition and timing.	Wilcoxon matched pairs test, effect size calculations.	ANOVA used to compare motor and cognitive timing performance.
(a) Report numbers of individuals at each stage of study, e.g., numbers potentially eligible, examined for eligibility, confirmed eligible, included in the study, completing follow-up, and analyzed. (b) Give reasons for non-participation at each stage. (c) Consider use of a flow diagram.	Participants categorized by CP severity, control-matching was performed.	Participant numbers tracked across intervention stages.	Flow of participants described, including matched controls.
(a) Give characteristics of study participants (e.g., demographic, clinical, social) and information on exposures and potential confounders. (b) Indicate number of participants with missing data for each variable of interest. (c) Cohort study—Summarize follow-up time (e.g., average and total amount).	Demographic data included CP diagnosis details, control group matched.	Participant movement characteristics analyzed quantitatively.	Data on participant demographics, including motor impairment levels.
Report numbers of outcome events or summary measures.	Reported outcome measures include reaction time and timing accuracy.	Outcome measures include changes in movement precision.	Timing accuracy and motor responses compared across groups.
(a) Give unadjusted estimates and, if applicable, confounder-adjusted estimates and their precision (e.g., 95% confidence interval). (b) Report category boundaries when continuous variables were categorized. (c) If relevant, consider translating estimates of relative risk into absolute risk for a meaningful time period.	Statistical analysis of unadjusted and adjusted estimates reported.	Precision estimates of movement timing improvements.	Timing errors and confidence intervals analyzed.
Report other analyses conducted, e.g., analyses of subgroups and interactions, and sensitivity analyses.	Comparison across subgroups analyzed for variability.	Subgroup analyses for different CP severity levels.	Sensitivity analysis for motor and cognitive components.
Summarize key results with reference to study objectives.	Findings summarized regarding inhibitory control and timing.	Results highlight improvements in motor synchronization.	Key results related to motor timing performance summarized.
Discuss limitations of the study, taking into account sources of potential bias or imprecision. Discuss both direction and magnitude of any potential bias.	Limitations include small sample size and cognitive variability.	Limitations related to small sample and lack of long-term data. No control group.	Study acknowledges bias in measuring motor execution timing.
Give a cautious overall interpretation of results considering objectives, limitations, multiplicity of analyses, results from similar studies, and other relevant evidence.	Results interpreted within cognitive processing theories.	Findings discussed in relation to neuroplasticity and timing.	Results interpreted within cognitive–motor control models.
Discuss the generalizability (external validity) of the study results.	Generalizability limited due to small clinical sample.	Study mainly relevant to rehabilitation contexts.	Limited generalizability to broader CP.
Give the source of funding and the role of the funders for the present study and, if applicable, for the original study on which the present article is based.	Funding sources mentioned.	Although the funding sources are mentioned, there is no explicit statement about the potential role of the funders in the study design, data analysis, or interpretation of the results.	The study does not include any statement about funding sources or conflicts of interest, leaving this information unreported

**Table 4:** AXIS tool risk of bias assessment for Cabezas & Carriedo, 2020 [21]; Johansson et al., 2014 [37]; Olivier et al., 2015 [22]. ● = Yes, ⸰ = No, ■ = Partially reported, □ = Not reported.

	Cabezas & Carriedo, 2020 [21]		Johansson et al., 2014 [37]		Olivier et al., 2015 [22]	
Item	Response	Comments	Response	Comments	Response	Comments
1. Were the aims/objectives of the study clear?	●		●		●	
2. Was the study design appropriate for the stated aim(s)?	●		●		●	
3. Was the sample size justified?	⸰	Sample size justification not provided explicitly.	⸰	No power analysis or justification for sample size provided	⸰	No explicit justification for the small sample size.
4. Was the target/reference population clearly defined? (Is it clear who the research was about?)	●		●		●	
5. Was the sample frame taken from an appropriate population base so that it closely represented the target/reference population under investigation?	●		●		●	
6. Was the selection process likely to select subjects/participants that were representative of the target/reference population under investigation?	⸰	Bias possible due to selection of participants with specific impairments.	⸰	Selection limited by sample size and inclusion of children with severe DCP	⸰	Selection may not generalize to all CP children due to limited criteria
7. Were measures undertaken to address and categorise non-responders?	□	No information on measures for non-responders.	□	No mention of non-responders or how they were handled	□	No mention of measures for non-responders
8. Were the risk factor and outcome variables measured appropriate to the aims of the study?	●		●		●	
9. Were the risk factor and outcome variables measured correctly using instruments/measurements that had been trialled, piloted or published previously?	●		●		●	
10. Is it clear what was used to determine statistical significance and/or precision estimates? (e.g., *p*-values, confidence intervals)	●		●		●	
11. Were the methods (including statistical methods) sufficiently described to enable them to be repeated?	●		●		●	
12. Were the basic data adequately described?	●		●		●	
13. Does the response rate raise concerns about non-response bias?	⸰	No mention of potential non-response bias.	⸰	No evidence of non-response bias mitigation	⸰	No explicit discussion of response rate concerns or potential bias
14. If appropriate, was information about non-responders described?	⸰	No description of non-responders provided.	⸰	No information about non-responders was included.	⸰	Non-responders not described in any section of the paper
15. Were the results internally consistent?	●		●		●	
16. Were the results presented for all the analyses described in the methods?	●		●		●	
17. Were the authors’ discussions and conclusions justified by the results?	●		●		●	
18. Were the limitations of the study discussed?	●		●		●	
19. Were there any funding sources or conflicts of interest that may affect the authors’ interpretation of the results?	●		■	Although the funding sources are mentioned, there is no explicit statement about the potential role of the funders in the study design, data analysis, or interpretation of the results.	□	Funding sources mentioned but roles not clarified
20. Was ethical approval or consent of participants attained?	●		●		●	

**Table 5:** ROB 2 tool risk of bias assessment for Janeslätt et al. (2015) [43].

D1 Risk of Bias Arising from the Randomization Process	D2 Risk of Bias Due to Deviations from the Intended Interventions (Effect of Assignment to Intervention)	D3 Missing Outcome Data	D4 Risk of Bias in Measurement of the Outcome	D5 Risk of Bias in Selection of the Reported Result	Overall
⏺	⏺	⏺	⏺	⏺	⏺
Some concerns	Some concerns	Some concerns	Some concerns	Some concerns	Some concerns

## Data Availability

Data will be available on request to the corresponding author.

## Abbreviations

The following abbreviations are used in this manuscript:

TP	Time perception
CP	Cerebral palsy
SMT	Synchronized metronome training
TPA	Time-processing ability
SMA	Supplemental motor area
BG	Basal ganglia
